# To assess the effective and safety of berberine hydrochloride in ulcerative colitis: A protocol for systematic review and meta-analysis

**DOI:** 10.1097/MD.0000000000023482

**Published:** 2020-12-04

**Authors:** Yong Zhang, Jin Wang, Daorui Hou, Shuguang Yan, Sijie Dang

**Affiliations:** a Department of Gastroenterology, Sichuan Second Chinese Medicine Hospital, Chengdu; b Chengdu University, Chengdu, Sichuan; c Shaanxi University of Chinese Medicine, Xianyang, Shaanxi; d Department of Traditional Chinese Medicine Oncology, The First People's Hospital of Xiangtan City, China.

**Keywords:** berberine hydrochloride, protocol, systematic review and meta-analysis, ulcerative colitis

## Abstract

**Background:**

Ulcerative colitis (UC) is an inflammatory bowel disease characterized by a relapsing and remitting course, and the curative medical therapy of UC is not yet available with its precise etiology unknown. Berberine hydrochloride, one of the main alkaloids in rhizomes of Coptis chinensis, has been reported the efficacy in patients with UC. However, there is no systematic review related to berberine hydrochloride for UC published. In this work, we will systematically evaluate the effectiveness and safety of berberine hydrochloride for UC by a meta-analysis method to provide a substantial conclusion for clinical practice.

**Methods and analysis:**

In this study, we will search the Chinese and English databases by electronic and manual search to find the related literature of berberine hydrochloride in the treatment of UC published from the inception date of each predefined database up to October 2020. Databases include PubMed, Embase, MEDLINE, Cochrane Library Central Register of Controlled Trials, China National Knowledge Infrastructure (CNKI) database, Wanfang Data Knowledge Service Platform, the VIP information resource integration service platform (cqvip), China Biology Medicine Disc (Sino Med), the Chinese Clinical Trial Registry (ChiCTR), and ClinicalTrials.gov. The 2 professional trained authors will independently select the qualified studies for data extraction and assess the risk of bias in included studies. Then the synthesis and analyses of data will be carried out in RevMan 5.4. The heterogeneity of statistics will be assessed by a heterogeneity *X*^*2*^ test and *I*^*2*^ tests. Sensitivity analysis is used to evaluate whether the outcomes of systematic review or meta-analysis are robust and reliable. The funnel plot is the main method to evaluate the bias of reporting. Finally, we will use The Grading of Recommendations Assessment, Development and Evaluation to evaluate the quality of evidence.

**Results:**

The results of this study will be published in a peer-reviewed journal.

**Conclusion:**

Whether berberine hydrochloride is an effectiveness and safety for patients with UC will be judged in the conclusion of this systematic review.

**OSF registration number:**

10.17605/OSF.IO/X57U3.

## 1 Introduction

Ulcerative colitis (UC) is an inflammatory bowel disease characterized by chronic mucosal inflammation of the colon, and classified as proctitis, left-sided colitis, and extensive colitis according to disease extent.^[1]^ The prevalence and incidence of UC have been steadily increasing around the world over recent decades, the highest incidence rate of UC is Europe, followed by North America, and Asia, and the Middle East again.^[2]^ On account of the high prevalence and high per-patient costs of UC, it has accounted for substantial costs to the health care system and society.^[3]^ Compared with the general population, the incidence rate of colorectal cancer increased 2.4 times in patients with ulcerative colitis,^[4]^ which is associated with disease duration, extent, and more severe or persistent inflammatory activity.^[1]^ The cause of UC may be associated with an interaction between genetic and environmental factors, and its precise etiology is unknown.^[5]^ So curative medical therapy is symptomatic treatment, and the therapy goals of UC are to maintain remission, cut down the risk of complications, and improve quality of life of patients. Choice of treatment is on the basis of the severity, distribution and pattern of disease. 5-aminosalicylic acid, corticosteroids, immunosuppressants, and anti-TNF therapy are recommendations for the Medical Management of UC.^[6]^ However, long-term drug use can cause many side effects,^[7]^ which has limited its clinical application.

Berberine hydrochloride is one of the main alkaloids in rhizomes of Coptis chinensis, a kind of traditional Chinese herb first recorded in Shennong herbal classic. There are diverse pharmacological effects of berberine hydrochloride including anti-inflammation,^[8,9]^ anticancer,^[10]^ lower elevated blood glucose,^[11–13]^ and immunoregulatory,^[14]^ which is wildly used in various diseases, especially in digestive system diseases.^[15]^ It also proved by the China Food and Drug Administration for the treatment of chronic gastroenteritis (approved No. Z20040003). Recently, more and more clinical studies have reported the efficacy of berberine hydrochloride in patients with UC in improving the clinical symptoms and quality of life of patients.^[16,17]^ The possible mechanism of berberine hydrochloride in treatment of UC is related to anti-inflammatory response by blocking the NF-κB signaling pathway^[18,19]^ and the inflammatory cytokines, such as TNF-α, IL-1β, IL-10.^[20]^ However, there is no a systematic review related to berberine hydrochloride for UC up to now. The effectiveness and safety of berberine hydrochloride on UC will be urgently support by a systematic review. In this work, we will systematically evaluate the effectiveness and safety of berberine hydrochloride for UC by a meta-analysis method, so as to provide a substantial conclusion for clinical practice.

## 2 Methods and analysis

### 2.1 Study registration

This protocol report has been registered at Open Science Framework (OSF, https://osf.io/), an open source project management for research and design. The registration DOI of this study is 10.17605/OSF.IO/X57U3. This protocol report is conducted according to the Preferred Reporting Items for Systematic Reviews and Meta-Analyses Protocols (PRISMA-P) statement guidelines.^[21]^

### 2.2 Inclusion and exclusion criteria

#### 2.2.1 Study design

The type of study design for this protocol will be limited to randomized controlled trials (RCTs), and animal mechanism studies, case reports, self-controlled studies, non-RCTs, randomized crossover studies, and quasi-randomized trials will be excluded. Language in the research will be limited to English and Chinese.

#### 2.2.2 Type of participants

All recruited participants are diagnosed with UC by medical history, clinical evaluation, endoscopic parameters, and histopathology, and careful exclusion of infectious colitis. There will be no limitation about age, gender, region, education, economic status, and other factors.

#### 2.2.3 Interventions/comparators

It will be included in our protocol about those studies in which interventions involved berberine hydrochloride alone or combined with other routine pharmacotherapy, and the control group includes placebo control, no treatment, and conventional treatments, such as 5-aminosalicylic acid, corticosteroids, and Chinese herbal compound. The method of administration can be oral or enema, and the treatment durations above studies should be 2 to 10 weeks.

#### 2.2.4 Outcomes

The primary outcomes of this review will focus on the mucosal healing (normal-looking mucosa with no significant inflammation) by endoscopy, and the secondary outcomes include the relief of clinical symptoms and Mayo endoscopic subscore.^[7]^ Any adverse events will also be included in the work.

### 2.3 Study search

In this study, we will search the Chinese and English databases by electronic and manual search to find the related literature of berberine hydrochloride in the treatment of UC published from the inception date of each predefined database upto October 2020. Databases include PubMed, Embase, MEDLINE, Cochrane Library Central Register of Controlled Trials, China National Knowledge Infrastructure (CNKI) database, Wanfang Data Knowledge Service Platform, the VIP information resource integration service platform (cqvip), China Biology Medicine Disc (Sino Med), the Chinese Clinical Trial Registry (ChiCTR), and ClinicalTrials.gov. At the same time, we will search other professional related resources as much as possible, such as Google scholar, Bing scholar, and Baidu scholar. The languages of the above literatures are limited to Chinese and English. Terms for retrieval include Idiopathic Proctocolitis, Ulcerative Colitis, Colitis Gravis, Inflammatory Bowel Disease (IBM), Ulcerative Colitis Type, Umbellatine, and Berberine hydrochloride. A retrieval strategy is based on the Cochrane Handbook guidelines.^[22]^ The search strategy that combines MeSH terms and free words was as follows:

1#: Search: ((((Colitis, Ulcerative [MeSH Terms]) OR Idiopathic Proctocolitis [Title/Abstract]) OR Ulcerative Colitis [Title/Abstract]) OR Colitis Gravis [Title/Abstract]) OR Inflammatory Bowel Disease, Ulcerative Colitis Type [Title/Abstract]

2#: Search: ((Berberine [MeSH Terms]) OR Umbellatine [Title/Abstract]) OR Berberine hydrochloride [Title/Abstract]

3#:Search:(((((((((randomized controlled trial[Title/Abstract]) OR RCT[Title/Abstract]) OR random[Title/Abstract]) OR randomly[Title/Abstract]) OR random allocation[Title/Abstract]) OR allocation[Title/Abstract]) OR randomized control trial[Title/Abstract]) OR controlled clinical trial[Title/Abstract]) OR clinical trial[Title/Abstract]) OR clinical study[Title/Abstract]

#1 and #2 and #3

### 2.4 Study selection

**Figure 1:**
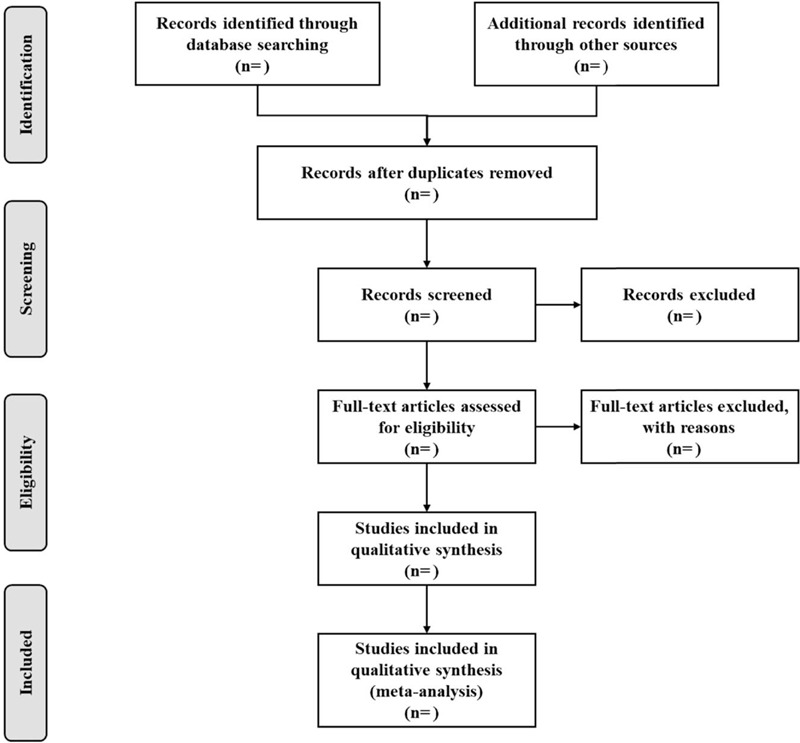
Flow chart of study selection.

It is necessary to carry out professional training for all reviewers, which can help them better understand the purpose of the review. The citations of electronic retrieval from the above databases will are imported into EndNote X9.0 (Stanford, Connecticut, https://endnote.com) to establish a database. Citations selected manually or from other resources will be included in the above database. Two authors (Yong Zhang and Sijie Dang) will independently read the titles and abstracts of the retrieved literatures to determine which literatures meet the established inclusion criteria. By reading the full text in detail, we will further review the literatures, of which the excluded literatures also be recorded and explained. When there are different opinions on the literatures, the 2 reviewers will solve the problem through negotiation. If necessary, we will contact the author of the paper for clarification. The procedure of study selection is shown in a PRISMA flow chart (Fig. 1).

### 2.5 Data extraction and management

Data extraction of those selected articles is realized with the 2 reviewers using Microsoft Excel to design the data collection form, which usually include the following information: the first authors, year of publication, country of publication, publication type, type of study, gender, age, interventions in experimental group and control group, time of treatment, ample size in each group, outcome indicators, and adverse events. In addition, we need to collect some other information, such as important citations, funding agencies, potential conflicts of interest, etc. When data are missing, or unclear and the methodological details are not described in papers, we will contact the author for more detailed information.

### 2.6 Assessment of risk of bias in included studies

Two reviewers will independently evaluate the risk of bias for all included studies by the Cochrane Collaboration's bias risk assessment tool, which includes the following aspects: random sequence generation (selection bias), allocation concealment (selection bias), blinding of participants and personnel (performance bias), blinding of outcome assessment (detection bias), incomplete outcome data (attrition bias), selective reporting (reporting bias), other bias. For each result of the above studies, we will make the judgment of “Low bias”, “High bias”, or “Unclear bias ”. If inconsistent results between the 2 reviewers occur, the final decision will be made by the third author.

### 2.7 Synthesis of data

The synthesis and analyses of data will be carried out in RevMan 5.4. If the data type to be analyzed is dichotomous variables, the odd ratio, relative risk, or risk difference can be selected. If it is continuous variables, a mean difference or a standard mean difference should be selected. The confidence intervals for the above 2 variables will be set to 95%.

### 2.8 Assessment of heterogeneity

According to the principle of statistics, only homogeneous data can be synthesized.

Therefore, it is necessary to test the heterogeneity of statistics assessed by a heterogeneity *X*^*2*^ test and *I*^*2*^ tests before the synthesis of data. If *P* > .10 and *I*^2^ ≤ 50%, it shows that the heterogeneity of multiple studies was not statistically significant. Then the fixed-effects model will be used to synthesize the data. If *P* ≤ .10 and *I*^2^ > 50%, the subgroup analysis will be used to analyze the causes of heterogeneity, such as the design scheme, measurement scheme, dosage, medication method, age, gender, course of disease, and other factors. If the heterogeneity still exists after using the above methods, the random effect model should be selected to calculate the effect size.

### 2.9 Sensitivity analysis

Sensitivity analysis is used to evaluate whether the outcomes of systematic review or meta-analysis are robust and reliable. We will re-analyze the data using different statistical methods, such as replacing the fixed effect model with a random effect model, and vice versa. If the sensitivity analysis has no essential change to the outcomes of systematic review or meta-analysis, the possibility of the analysis results will be greatly increased. On the contrary, it means that we should treat the result explanation and conclusion with caution.

### 2.10 Assessment of reporting biases

The common reporting biases can be divided into the following categories: publication bias, time lag bias, duplication publication bias, location bias, citation bias, language bias, outcome reporting bias, and so on. The funnel plot that should be regarded as a general method to show the small-study-effects, is the main method to evaluate the bias of reporting. When more than ten studies are included, Egger tests will be performed to assess the asymmetry of the funnel plot. A value of *P* < .05 will be considered to have a significant publication bias.^[23]^

### 2.11 Grading the quality of evidence

To assess the quality of evidence for the results obtained, we will use The Grading of Recommendations Assessment, Development and Evaluation (GRADE), which is a widely used tool in evaluating the quality of assessment.^[24]^ According to the research design methodology, the quality of evidence is divided into 4 levels: “high”, “moderate”, “low”, and “very low”.

### 2.12 Patient and public involvement

There are no patient and public involving in this study.

### 2.13 Ethics and dissemination

Since the data extracted in this study are not linked to individual patient, ethical approval is not necessary. The purpose of the study is to provide clinicians with evidence-based medical support about the treatment of berberine hydrochloride for UC, and the results of the study will be published in a peer-reviewed journal.

## 3 Discussion

Although UC is still a rare disease in China, the number of patients has been increasing rapidly in recent 20 years.^[25]^ There is not a “gold standard” for diagnosis of ulcerative colitis, which is established by medical history, clinical evaluation, endoscopic parameters, and histopathology, and careful exclusion of infectious colitis.^[5]^ The leading symptom of UC include bloody diarrhea, rectal bleeding, tenesmus, urgency, fecal incontinence, nocturnal defecation, fatigue, abdominal pain, anorexia, and fever,^[5]^ which are important determinants of health related quality of life.^[7]^ Many studies have shown that berberine hydrochloride is effective in relieving UC.^[26]^ However, there is no systematic review on this topic have been published, which limits the clinical application of berberine hydrochloride for UC. Therefore, it is necessary to conduct a systematic review and meta-analysis of the relevant studies published. In this work, we will systematically evaluate the effectiveness and safety of berberine hydrochloride for UC to provide clinicians with more supportive evidence in the decision-making process for UC treatment.

### 3.1 Amendments

If the protocol is needed to modify in the process of research, we will update the information in the final report.

## Author contributions

**Conceptualization:** Yong Zhang, Sijie Dang.

**Data curation:** Yong Zhang, Jin Wang, Sijie Dang.

**Formal analysis:** Jin Wang.

**Funding acquisition:** Sijie Dang, Shuguan Yan.

**Investigation:** Yong Zhang, Sijie Dang.

**Methodology:** Yong Zhang, Jin wang, Shuguan Yan, Sijie Dang.

**Project administration:** Sijie Dang.

**Resources:** Yong Zhang, Jin Wang, Daorui Hou, Shuguan Yan, Sijie Dang.

**Software:** Yong Zhang, Sijie Dang.

**Supervision:** Jin Wang, Shuguan Yan.

**Writing - original draft:** Yong Zhang.

**Writing – review & editing:** Jin wang, Sijie Dang.
